# Rimonabant Improves Oxidative/Nitrosative Stress in Mice with Nonalcoholic Fatty Liver Disease

**DOI:** 10.1155/2015/842108

**Published:** 2015-05-11

**Authors:** Bojan Jorgačević, Dušan Mladenović, Milica Ninković, Milena Vesković, Vesna Dragutinović, Aleksandar Vatazević, Danijela Vučević, Rada Ješić Vukićević, Tatjana Radosavljević

**Affiliations:** ^1^Institute of Pathophysiology “Ljubodrag Buba Mihailović”, Faculty of Medicine, University of Belgrade, 11000 Belgrade, Serbia; ^2^Institute for Medical Research, Military Medical Academy, 11000 Belgrade, Serbia; ^3^Institute of Medical Chemistry, Faculty of Medicine, University of Belgrade, 11000 Belgrade, Serbia; ^4^Faculty of Chemistry, University of Belgrade, 11000 Belgrade, Serbia; ^5^Institute of Digestive Diseases, Clinical Center of Serbia, 11000 Belgrade, Serbia

## Abstract

The present study deals with the effects of rimonabant on oxidative/nitrosative stress in high diet- (HFD-) induced experimental nonalcoholic fatty liver disease (NAFLD). Male mice C57BL/6 were divided into the following groups: control group fed with control diet for 20 weeks (C; *n* = 6); group fed with HFD for 20 weeks (HF; *n* = 6); group fed with standard diet and treated with rimonabant after 18 weeks (R; *n* = 9); group fed with HFD and treated with rimonabant after 18 weeks (HFR; *n* = 10). Daily dose of rimonabant (10 mg/kg) was administered to HFR and R group by oral gavage for two weeks. Treatment induced a decrease in hepatic malondialdehyde concentration in HFR group compared to HF group (*P* < 0.01). The concentration of nitrites + nitrates in liver was decreased in HFR group compared to HF group (*P* < 0.01). Liver content of reduced glutathione was higher in HFR group compared to HF group (*P* < 0.01). Total liver superoxide dismutase activity in HFR group was decreased in comparison with HF group (*P* < 0.01). It was found that rimonabant may influence hepatic iron, zinc, copper, and manganese status. Our study indicates potential usefulness of cannabinoid receptor type 1 blockade in the treatment of HFD-induced NAFLD.

## 1. Introduction

Nonalcoholic fatty liver disease (NAFLD) includes a wide spectrum of liver diseases ranging from simple steatosis to steatohepatitis, which can progress to fibrosis, cirrhosis, and, ultimately, hepatocellular carcinoma [1]. NAFLD, as a hepatic manifestation of metabolic syndrome, is associated with dyslipidemia, obesity, insulin resistance, and type 2 diabetes mellitus [1, 2]. Although pathogenesis of NAFLD is still incompletely understood, two key mechanisms have been identified. Firstly, insulin resistance in the adipose tissue increases lipolysis and the hepatic entry of free fatty acids, as well as* de novo* synthesis of fatty acids and triglycerides. Secondly, oxidative/nitrosative stress, mitochondrial dysfunction, lipotoxicity, and overproduction of proinflammatory cytokines have been involved in progression of liver steatosis to steatohepatitis and fibrosis [3, 4].

Excessive oxidants (free oxygen and nitrogen radicals, reactive oxygen species (ROS), reactive nitrogen species (RNS), etc.) and decreased antioxidant defense have been observed in NAFLD patients [2, 5, 6]. Thus, the main sources of ROS are hepatic microsomal fatty acid oxidizing enzyme cytochrome P450 2E1 (CYP2E1), cyclooxygenase and lipoxygenase cell signal transduction pathways, mitochondrial dysfunction, and hepatic iron overload [4, 7]. Moreover, activation of Kupffer cells and other inflammatory cells also generates ROS through nicotinamide adenine dinucleotide phosphate (NADPH) oxidase [8].

Endocannabinoid system (ECS) is a signaling cascade consisting of cannabinoid receptors (G-protein-coupled cannabinoid receptor type 1 /CB1/ and type 2 /CB2/), endocannabinoids (derivatives of arachidonic acid,* N*-arachidonoyl ethanolamine, also known as anandamide /ANA/, and 2-arachidonoyl glycerol /2-AG/), and several enzymes involved in the synthesis and degradation of endocannabinoids [9–13].

The presence of CB1 receptors in the liver has been confirmed, and both ANA and 2-AG have been detected in different types of liver cells, including hepatocytes and stellate cells [14–17]. A number of studies show that ECS is important in NAFLD pathogenesis and other obesity-related metabolic disorders, due to its crucial role in the regulation of metabolism and energy homeostasis [9, 11, 16, 18–20]. Thus, dysregulation of ECS in human obesity leads to increasing hedonic and homeostatic food intake, weight gain, and triglyceride accumulation in the liver and adipose tissue, with consequent hepatic steatosis and insulin resistance [9, 15, 18, 20, 21]. Apart from modulating lipid metabolism, the ECS modulates glucose metabolism by promoting pancreatic hormonal secretion that in turn favours glucose uptake by tissues such as the liver and white adipose tissue, leading to fatty acid synthesis and energy storage [13]. Additionally, ECS is involved in modulating the immune and inflammatory responses [11, 22–25].

The liver is identified as a primary site for endocannabinoid-mediated modulation of lipogenesis [26]. In fact, the activation of the CB1 receptor increases the expression of lipogenic genes in the liver, which is the main source of* de novo* fatty acid synthesis in the body [15, 17, 26]. It is suggested that hepatic CB1 receptors are implicated in the development of fatty liver, hypertriglyceridaemia, and other metabolic abnormalities in diet-induced obesity [12, 13, 17, 20, 26–30]. It is reported that CB1 receptor antagonism may protect the liver from high fat diet- (HFD-) induced phenomena such as hepatic steatosis [31–33], insulin resistance [26, 33], and glucose intolerance [13]. Having in mind that deletion of CB1 receptors in the liver decreases hepatic lipogenesis and ameliorates hypercholesterolemia, hypertriglyceridemia, and hepatocellular damage [12, 15], it has been hypothesized recently that the hepatic ECS may represent a target for the treatment of NAFLD [11, 34].

The ability of ECS to promote oxidative/nitrosative stress production and modulate antioxidant capacity in kidney and cardiac tissue has received great attention [25, 35, 36]. It is also suggested that inhibition of CB1 receptors may exert beneficial effects in renal diseases associated with oxidative/nitrosative stress [25]. However, at present nothing is known about the precise role of ECS in initiation and propagation of oxidative/nitrosative hepatic injury. Based on this background, the aim of the present study was to investigate the effect of CB1 receptor blockade on oxidative/nitrosative stress in mice with NAFLD.

## 2. Materials and Methods

### 2.1. Animals

The experiment was performed on male mice C57BL/6 weighting 21–25 g (8 weeks) that were raised on Military Medical Academy in Belgrade. Animals were kept in individual cages under standard laboratory conditions (ambient temperature 22 ± 2°C, relative humidity 50 ± 10%, and 12/12 h dark/light cycle with lights turned on at 9.00 AM) with free access to tap water and standard chow diet. Daily food intake was measured during the experiment. All experimental procedures were in full compliance with Directive of the European Parliament and of the Council (2010/63EU) and were approved by the Ethical Committee of the University of Belgrade (Permission No 695/2).

### 2.2. Experimental Design

All animals (*n* = 31) were randomly divided into the following groups: (1) control group fed with control chow diet for 20 weeks (C; *n* = 6); (2) group fed with high saturated fat diet for 20 weeks (HF; *n* = 6); (3) group fed with standard chow diet and treated with rimonabant after 18 weeks (R; *n* = 9); (4) group fed with high saturated fat diet and treated with rimonabant after 18 weeks (HFR; *n* = 10). Composition of high saturated fat diet (MP Biochemicals, CA, USA) is shown in Table 1. Daily dose of rimonabant (10 mg/kg) was administered to HFR and R group by oral gavage for two weeks [37]. Simultaneously, C and HF group received vehicle (saline, 0.9% NaCl) in the same manner. Before administration, rimonabant was dissolved into 0.1% Tween 80 in distilled water and for 20 s sonificated on ice using a digital Branson sonificator.

Mice were fasted overnight and after that they were sacrificed by exsanguination in ketamine (100 mg/kg intraperitoneally /i.p./) anesthesia. Liver samples were taken for pathohistology analysis and determination of oxidative/nitrosative stress parameters and oligoelement levels.

### 2.3. Sample Preparation

Liver samples for biochemical analysis were homogenized on ice, in cold buffered 0.25 M sucrose medium (Serva, Feinbiochemica, Heidelberg, New York), 10 mM phosphate buffer (pH 7.0), and 1 mM ethylenediaminetetraacetic acid (EDTA, Sigma chem. Co., St. Louis, USA). The homogenates were centrifuged at 2000 ×g for 15 minutes at 4°C. Crude sediments were dissolved in a sucrose medium and centrifuged. The supernatants were transferred into the tubes and centrifuged at 3200 ×g for 30 minutes at 4°C. Obtained sediments were dissolved in deionized water. After one hour of incubation, the samples were centrifuged at 3000 ×g for 15 minutes at 4°C, and supernatants were stored at −70°C. Proteins were determined by the Lowry method using bovine serum albumin as the standard [38].

### 2.4. Biochemical Parameters

#### 2.4.1. Determination of Hepatic Oxidative/Nitrosative Stress Parameters

Lipid peroxidation, measured as malondialdehyde (MDA) level, was determined spectrophotometrically in a reaction with thiobarbituric acid as described by Girotti et al. [39]. The results are expressed as nmol of MDA per milligram of proteins (nmol/mg protein).

The concentration of nitrites + nitrates (NO_*x*_) as a measure of NO production was determined by using Griess reagent. After reduction of the nitrates, the total nitrites were reacted with sulfanilamide and* N*-(1-naphthyl) ethylenediamine to produce an azo dye, which was measured spectrophotometrically at 492 nm [40].

Activity of total superoxide dismutase (EC1.15.1.1.; SOD) in the liver was measured spectrophotometrically, as an inhibition of epinephrine autooxidation at 480 nm. After addition of 10 mM epinephrine (Sigma, St. Louis, USA), analysis was performed in the sodium carbonate buffer (50 mM, pH 10.2; Serva, Feinbiochemica, Heidelberg, New York) containing 0.1 mM EDTA (Sigma, St. Louis, USA). Samples for manganese SOD (MnSOD) were previously treated with 8 mM KCN (Sigma, St. Louis, USA) and then analysed as previously described [41]. Activity of copper/zincSOD (Cu/ZnSOD) was determined as difference between the activities of total SOD and MnSOD. All enzyme activities are expressed as units per milligram of protein (U/mg prot.).

The content of reduced glutathione (GSH) was determined spectrophotometrically using 5,5-dithio-bis-2-nitrobenzoic acid (DTNB). DTNB reacts with aliphatic thiol compounds at pH 8.0 forming yellow p-nitrophenol anion whose absorption is measured spectrophotometrically at 412 nm [42]. Results are expressed as nmol per milligram of proteins (nmol/mg protein).

#### 2.4.2. Determination of Oligoelement Contents in the Liver

A MDS-2000 system microwave digestion lab station was used for digesting the samples of tissue with 10 mL of diluted nitric acid (1 : 1), followed with addition of 10 mL concentrated nitric acid. Samples were treated according to the program of 3 steps. This was done over the period of 20 min with the power of 630 ± 30 W and within the temperature range of 140–160°C. After complete digestion samples were transferred in 50 mL volumetric flask [43].

Zn and Cu liver content was determined by means of flame atomic absorption spectrometry (FAAS), while graphite furnace atomic absorption spectrometry (GF AAS) was applied for determining of Mn and iron (Fe) liver content [43].

Oligoelement liver content is expressed as milligram per kilogram (mg/kg).

All chemicals for the digestion of the samples were of the analytical reagent grade and were supplied by J.T. Baker Chemical Co. (Phillipsburg, NJ, USA).

### 2.5. Pathohistological Analysis

Liver tissue was incubated in 10% formalin solution at room temperature. After the fixation, the liver samples were processed by the standard method. Tissues were incorporated in paraffin sectioned at 5 *μ*m and then stained with Hematoxylin-Eosin (HE) and prepared for light microscopy analysis. All samples were evaluated by an experienced pathohistologist who was blinded to the experiment. Preparations were analyzed and photographed using a combined photobinocular light microscope Olympus BX51 equipped with Artcore 500MI artery, Co. Ltd. Japan Camera.

### 2.6. Statistical Analysis

All results are expressed as means ± SD. As the normal distribution of parameters was confirmed by Kolmogorov-Smirnov test, one-way analysis of variance (ANOVA) with Tukey's post hoc test was used for testing the difference among groups. The difference was considered significant if *P* < 0.05. For statistical analysis computer software SPSS 15.0 was used.

## 3. Results

### 3.1. Effect of Rimonabant on Liver/Body Weight Ratio

There was no statistically significant difference in liver/body weight ratio between C group (4.65 ± 0.33%) and R group (4.54 ± 0.41%) (*P* = 0.955), as well as in HF group (4.86 ± 0.44%) and HFR group (4.67 ± 0.38%) (*P* = 0.907).

### 3.2. Effect of Rimonabant on Food Intake

**Figure 1:**
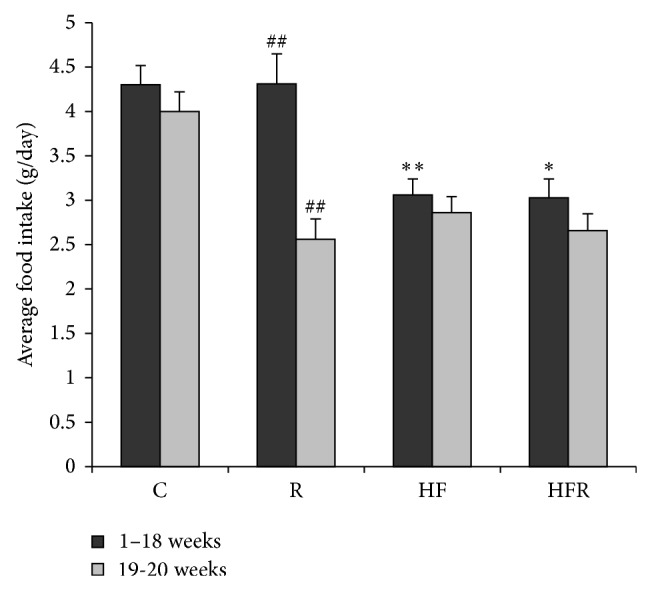
The effects of control chow and high fat diet on food intake during 20 weeks of treatment (C, HF, R, and HFR). Statistical significance of the difference was estimated by using one-way analysis of variance (ANOVA) with Tukey's post hoc test (^∗^ *P* < 0.05 versus first 18 weeks; ^∗∗^ *P* < 0.01 versus first 18 weeks; ^##^ *P* < 0.01 versus control group).

Food intake was significantly higher in group fed with control chow diet (4.30 ± 0.22 g) compared to HFD fed mice (3.06 ± 0.18 g) (*P* = 0.004). Treatment with rimonabant induced a significant decrease in food intake in HFR group in 19th and 20th weeks (2.66 ± 0.19 g resp.) compared to first the 18 weeks (3.03 ± 0.21 g) (*P* = 0.033). Similarly, decrease in food intake was observed in R group in 19th and 20th weeks (2.56 ± 0.23 g) compared to the first 18 weeks (4.31 ± 0.34 g) (*P* = 0.002) (Figure 1).

### 3.3. Effect of Rimonabant on Hepatic Oxidative/Nitrosative Stress Parameters

**Figure 2:**
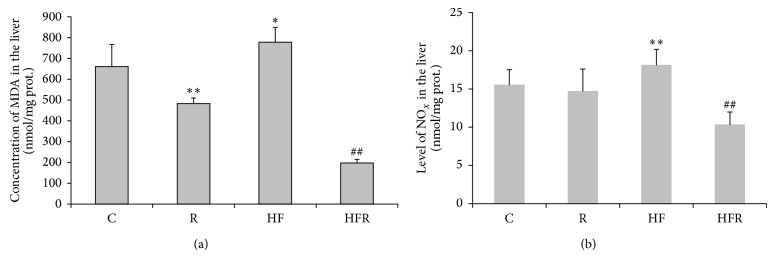
The effect of rimonabant on the level of malondialdehyde (MDA) in the mice liver after 20 weeks feeding with high fat and control chow diet (a). The effect of rimonabant on the NO_*x*_ (nitrites + nitrates) in the mice liver after 20 weeks feeding with high fat and control chow diet (b). Statistical significance of the difference was estimated by using one-way analysis of variance (ANOVA) with Tukey's post hoc test (^∗^ *P* < 0.05, ^∗∗^ *P* < 0.01 versus C, and ^##^ *P* < 0.01 versus HF).

MDA concentration was significantly increased in HF group (777.99 ± 70.87 nmol/mg prot.) compared to control (661.09 ± 106.72 nmol/mg prot.) (*P* = 0.023). On the other hand, rimonabant treatment induced a significant decrease in MDA concentration in HFR group (197.04 ± 17.99 nmol/mg prot.) in comparison with HF group (*P* = 0.001). Significant decrease in MDA concentration was also observed in control chow diet fed mice treated with rimonabant (483.18 ± 26.59 nmol/mg prot.) compared to control (*P* = 0.001) (Figure 2(a)).

HFD caused an increase in NO_*x*_ content (18.10 ± 2.94 nmol/mg prot.) compared to control (15.51 ± 2.02 nmol/mg prot.) (*P* = 0.007). Rimonabant treatment induced a significant decrease in NO_*x*_ concentration in HFR group (10.31 ± 1.66 nmol/mg prot.) in comparison with HF group (*P* = 0.001) (Figure 2(b)).

**Figure 3:**
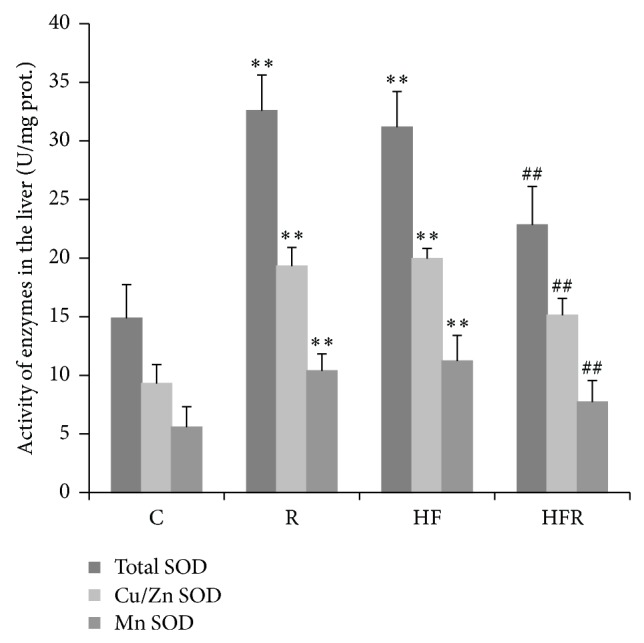
The effect of rimonabant on total SOD, Cu/ZnSOD, and MnSOD activity in the liver after 20 weeks feeding with high fat and control chow diet. Statistical significance of the difference was estimated by using one-way analysis of variance (ANOVA) with Tukey's post hoc test (^∗^ *P* < 0.05,^∗∗^ *P* < 0.01 versus C, and^ ##^ *P* < 0.01 versus HF).

Activity of total SOD was significantly increased in HF group (31.17 ± 3.05 U/mg prot.) compared to C group (14.88 ± 2.86 U/mg prot.) (*P* = 0.001). Besides, higher total SOD activity was registered in R group (32.57 ± 3.06 U/mg prot.) compared to control (*P* = 0.001). On the other hand, total SOD activity in HFR group (22.84 ± 3.27 U/mg prot.) was significantly decreased in comparison with HF group (*P* = 0.004). Analysis of SOD isoenzymes revealed that expression of MnSOD and Cu/ZnSOD followed the changes of total SOD activity in all groups (Figure 3).

**Figure 4:**
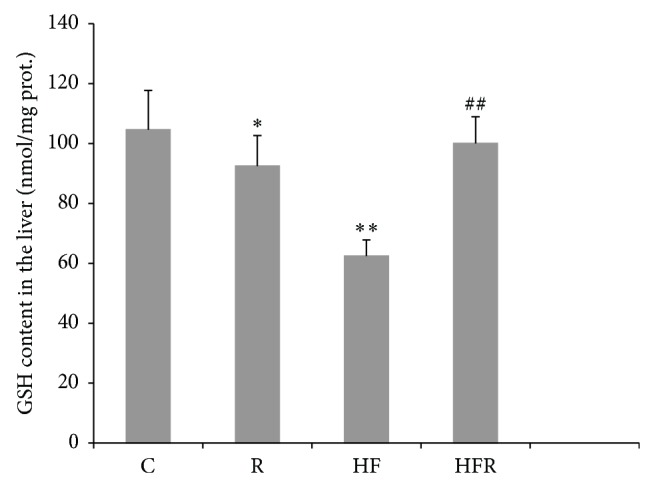
The effect of rimonabant on the GSH content in the mice liver after 20 weeks feeding with high fat and control chow diet. Statistical significance of the difference was estimated by using one-way analysis of variance (ANOVA) with Tukey's post hoc test (^∗^ *P* < 0.05, ^∗∗^ *P* < 0.01 versus C, and ^##^ *P* < 0.01 versus HF).

GSH content was significantly decreased in HF group (62.43 ± 8.86 nmol/mg prot.) in comparison with control (104.59 ± 13.16 nmol/mg prot.) (*P* = 0.003). However, GSH level was significantly higher in HFR group (100.09 ± 5.39 nmol/mg prot.) compared to HF group (*P* = 0.009). Besides, decrease in GSH concentration was observed in R group (92.46 ± 10.19 nmol/mg prot.) when compared to control (*P* = 0.041) (Figure 4).

### 3.4. Effect of Rimonabant on Liver Oligoelement Contents

It was found a significant decline in Zn content in HF group (29.10 ± 2.38 mg/kg) in comparison with C group (36.69 ± 5.02 mg/kg) (*P* = 0.033). However, Zn content in HFR group (50.1 ± 8.65 mg/kg) was higher compared to HF group (*P* = 0.001) and also in R group (45.79 ± 6.16 mg/kg) compared to C group (*P* = 0.009) (Table 2).

It was shown a significant HFD-induced increase in Fe content (110.59 ± 10.07 mg/kg) when compared to control (82.90 ± 4.83 mg/kg) (*P* = 0.004). In contrast, treatment with rimonabant reduced Fe content in HFR (70.46 ± 10.07 mg/kg) in comparison with HF group (*P* = 0.003) (Table 2).

Cu content in HF group (4.33 ± 0.24 mg/kg) was significantly increased in comparison with control (2.66 ± 0.15 mg/kg) (*P* = 0.001). Significant increase in Cu content was also observed in HFR group (5.53 ± 1.38 mg/kg) compared to HF group (*P* = 0.005), as well as in R group (4.04 ± 0.48 mg/kg) in comparison with C group (*P* = 0.001) (Table 2).

Similarly to Cu content, HFD induced an increase in Mn content (2.41 ± 0.23 mg/kg) compared to control value (1.27 ± 0.01 mg/kg) (*P* < 0.003). Administration of rimonabant increased Mn content in HFR group (10.01 ± 1.99 mg/kg) in comparison with HF group, too (*P* < 0.001). In contrast to Cu content, Mn content in R group (0.06 ± 0.01 mg/kg) was decreased when compared to C group (*P* < 0.001) (Table 2).

### 3.5. Pathohistological Findings

**Figure 5:**
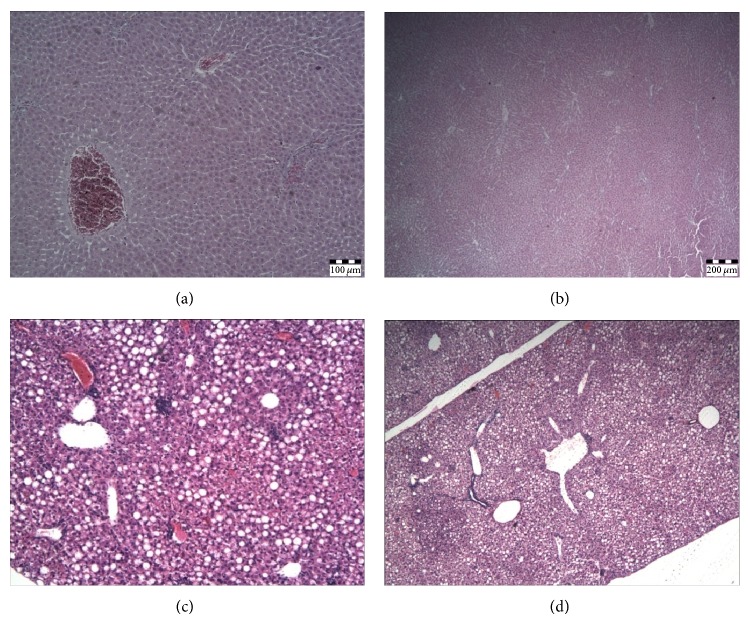
The effect of HF diet on histopathological changes in the liver. HE-stained preparations were sectioned with blade thickness of 5 *μ*m and analyzed and photographed using a combined photobinocular light microscope Olympus BX51. (a) Control with normal liver histology /10x/; (b) rimonabant treated control chow diet fed group with normal liver histology /10x/; (c) liver histology in HF group shows mild steatosis with portal inflammatory infiltrate and focal necrotic changes in parenchyma /20x/; (d) liver histology in HFR group shows mild steatosis /10x/.

There were no pathohistological changes in the liver in C and R groups (Figure 5(a) and 5(b)). HFD caused mild hepatic steatosis with portal inflammatory infiltrate and focal necrotic changes in parenchyme (Figure 5(c)). In contrast to HF group, mild steatosis and no inflammatory infiltrate in portal space and focal necrotic changes in the liver parenchyma were found in HFR group (Figure 5(d)).

## 4. Discussion

Results of our study clearly showed that HFD induced lipid peroxidation and nitrosative stress in the liver that are associated with steatosis and portal inflammation (Figure 5(c)). The role of oxidative stress in the pathogenesis of NAFLD is well known and is bidirectionally linked with inflammation [5–8]. Lipid peroxidation along with cytokine production and Fas ligand induction causes hepatocyte injury and apoptosis [7]. Besides, activated Kupffer cells and other inflammatory cells produce ROS and aggravate hepatic lipid peroxidation [8]. Additional sources of ROS in NAFLD include CYP2E1 induction by free fatty acids [4, 44], mitochondrial dysfunction, and increased NADPH oxidase activity [44, 45], as well as accumulation of ceramides within the hepatocytes [46]. To eliminate higher HFD food intake as a possible reason for ROS hyperproduction within the hepatocytes, we have measured food intake daily. In our study food intake could not be contributing factor which influenced level of hepatic oxidative/nitrosative stress (Figures 1 and 2).

Our study suggests that endocannabinoids also aggravate oxidative stress in NAFLD and contribute to the development of nonalcoholic steatohepatitis (NASH) through CB1 receptors. In fact, this is the first study that deals with effects of endocannabinoids and the selective CB1 antagonist, rimonabant, on oxidative/nitrosative stress in the liver. Namely, prooxidative effects of endocannabinoids are suggested by reduction of lipid peroxidation in both standard diet- and HFD-fed animals after rimonabant treatment (Figure 2). However, the effect of rimonabant was more prominent in HFR group and was associated with reduction of inflammation (Figure 5(d)). Previous preclinical and clinical cardiomiopathy [35] and nephropathy [25] studies have shown anti-inflammatory and cytoprotective effects of CB1 antagonists, due to reduction of tumor necrosis factor-alpha (TNF-*α*) production and inactivation of nuclear factor-kappaB (NF-*κ*B). Similarly, our study clearly suggests that anti-inflammatory effect of rimonabant may be additionally mediated by decline in lipid peroxidation. Besides, this finding is in accordance with higher level of ANA and upregulation of CB1 receptor in HFD-induced NAFLD [20].

In present study, HFD caused an increase in NO_*x*_ content compared to control (Figure 2(b)). This finding is in agreement with reports demonstrating a link between HFD and nitrosative liver injury [45, 47–52]. It is known that an increased dietary supply of fat to the liver may promote steatosis by increasing hepatic lipid uptake. In this pathway, oxidative/nitrosative stress appears to play a key role in the pathogenesis of NASH [52], and nitric oxide (NO) may potentiate cytotoxicity through its reaction with superoxide anion and formation of nitrotyrosine [47, 48]. Furthermore, there is a relationship between abnormal hepatic accumulation of nitrotyrosine and pathohistological severity of NASH [47]. Besides, a strong expression of inducible nitric oxide synthase (iNOS) is observed in the liver from mice fed a HFD [52]. In addition, in our study inflammation could contribute to nitrosative stress in HF group. It is well established that hepatic inflammation is a complicated condition that is caused by various factors, including oxidative/nitrosative stress [2]. In such circumstances, activation of inflammatory cells in the liver generates ROS and RNS. Thus, peroxynitrite as a potent RNS not only can react with membrane phospholipids, DNA, and proteins, but also can produce cell damage via indirect radical-mediated mechanisms [53]. Although oxidative/nitrosative stress may not initiate hepatic inflammation, ROS and RNS overproduction could cause hepatocyte injury or death and, in turn, cytokine release that provides positive feedback on inflammatory signaling and promotes NASH pathogenesis [2]. It has revealed that NF-*κ*B is essential for inflammatory cell recruitment in NASH [54] and induction of the iNOS gene in different cell types including macrophages [52, 54]. Also, there is new evidence that HFD may lead to mitochondrial dysfunction and increased oxidative/nitrosative stress in liver and skeletal muscle [45, 50, 51].

The induction of iNOS in the liver seemed to correlate with fatty changes. Namely, the expression of iNOS is upregulated by most inducers of obesity, hyperglycemia, and insulin resistance, including free fatty acids [49, 52]. However, it is not clear how iNOS in liver with steatohepatitis interacts to expand hepatic lipid stores, causes hepatocellular injury, and recruits inflammation [52]. Our study indicates that rimonabant treatment induced a significant decrease in NO_*x*_ content in HFR group in comparison with HF group (Figure 2). Possible explanation for this result refers to anti-inflammatory and cytoprotective effects of CB1 pharmacological inhibition or genetic deletion observed in the numerous preclinical and clinical reports [9, 11–13, 15–21, 23–29, 31, 33–36]. Since increased oxidative/nitrosative stress and inflammation are known to trigger increased endocannabinoid production or impair endocannabinoid inactivation [55–57], it is likely that endocannabinoids contribute to HFD-induced liver damage in present experimental model of NAFLD by promoting expression of iNOS in hepatic parenchymal cells and/or peripheral blood mononuclear cells through the activation of CB1 receptors [58–61]. Additionally, our result may be related to published data that highlight the importance of CB1 receptor blockade in protection against the pathological consequences of a HFD in the liver [11, 16, 20, 25–27, 29].

GSH, as a multifunctional, intracellular, nonenzymatic antioxidant, is the major component of intracellular regulation of the redox state and provides the first line of defense against oxidative/nitrosative injury. Moreover, GSH is the important substrate and cofactor in drug metabolism [62]. However, abnormally large concentrations of ROS/RNS may lead to permanent changes in signaling mechanisms that respond to alterations in the thiol/disulfide redox state [63]. Our study demonstrated that HFD induced a significant depletion in GSH liver content (Figure 4). Consistent with previous reports [2, 6, 45, 48, 50–52], this finding indicates that HFD shifts the redox state towards prooxidant. Additionally, depletion in GSH liver content obtained from current study is accompanied with increased lipid oxidative damage (Figure 2) and taken together they confirmed the existence of oxidative/nitrosative stress in mice treated with HFD [52]. Besides, possible iNOS suppression may additionally decrease GSH level in the liver, having in mind that GSH can conjugate with NO to form a S-nitroso-glutathione [64, 65].

The results of our investigation found that liver GSH level was significantly higher in HFD fed mice treated with rimonabant compared to HFD fed mice, as well as in rimonabant treated mice in comparison with control (Figure 4). This rise in GSH level can be explained by an adaptive response of hepatocytes to increased ROS production. It also suggests that CB1 receptor blockade could have a beneficial effect on the redox state in hepatocytes.

SOD, as the principal enzyme in the antioxidant defense of cells, is involved in the conversion of superoxide anion into a less toxic hydrogen peroxide. It is present in hepatocytes in two isoforms, Cu/ZnSOD (cytosolic SOD) and MnSOD (mitochondrial SOD) [7]. In our study an increase in total hepatic SOD activity was observed in HFD fed mice (Figure 3). In addition, the activity of both isoforms of this enzyme was found to be increased in response to HFD treatment (Figure 3). This rise may be interpreted as an adaptive response of hepatocytes to increased ROS production induced by HFD. These findings correspond to the results of other studies indicating that liver is the organ with most prominent increase in the activity of SOD after exposure to HFD [4, 6, 8, 45, 50–52]. An increase in the activity of MnSOD obtained in our study is not surprising, since mitochondria are the most important organelles in ROS production [45]. Moreover, MnSOD has ability to limit the peroxynitrite formation [66].

In present study higher activity of total SOD and its isoenzymes was registered in liver of rimonabant-treated mice (Figure 3). Given the role of SOD, as a major defense system against superoxide, it seems that increase in its activity upon the action of rimonabant improved antioxidant defense system in the liver and makes hepatocytes less sensitive to oxidative/nitrosative damage. This result is similar to the documented data which reports that blockade of CB1 receptors may exert beneficial effects in renal and cardiac diseases associated with oxidative/nitrosative stress [25, 35, 36].

In the present study total hepatic SOD activity and its isoenzymes were significantly decreased in HFD fed mice treated with rimonabant in comparison with mice treated with HFD (Figure 3). This decrease of total hepatic SOD activity, as well as depletion in both mitochondrial and cytosolic SOD activity, indicates that blockade of CB1 receptors may modulate liver antioxidant capacity without influence on SOD activity.

In the current study, HFD caused an increase in iron liver content when compared to control values (Table 2). This result is compatible with most reports which support the hypothesis that interactions between hepatic iron and lipid metabolism play a role in NASH/NAFLD pathophysiology [67–70]. Accordingly, iron is an integral part of some enzymes and transporters involved in lipid metabolism and, as such, may exert a direct effect on hepatic lipid load, intrahepatic metabolic pathways, and hepatic lipid secretion. On the other hand, iron in its ferrous form may indirectly affect lipid metabolism. Namely, iron overload can generate oxidative stress and lipid peroxidation, which may modify the fatty acid profile of cellular membranes, leading to their disruption, damage to cell organelles, and impairment of mitochondrial oxidative metabolism [68]. It is suggested that the free radicals that form may cause a change in the ratio of saturated to unsaturated membrane phospholipids, leading to alterations in membrane fluidity [63]. This in turn may affect the activity of the embedded enzymes, so they respond to oxidative stress differently by altering their activity [71]. Besides, iron accumulation has a proinflammatory and profibrogenic role by activating Kupffer cells to release inflammatory cytokines and by activating hepatic stellate cells, which can culminate in the replacement of parenchymal tissue with connective tissue [72].

Our study has shown that administration of rimonabant to HFD fed mice reduced hepatic iron content in comparison with mice fed with HFD (Table 2). Since upon high fat feeding iron overload and CB1 receptors may be implicated in hepatocellular injury, our finding suggests that beneficial effects of CB1 receptor antagonism on iron-associated oxidative stress may result from improvement of iron metabolism disturbances.

The result of the present study was found a significant decline in zinc content in mice fed with HFD compared to control values (Table 2). This decline is in agreement with published data that indicate the ability of zinc to modulate hepatic gene expression and lipid homeostasis [73]. Zinc finger transcription factors, peroxisome proliferation activator receptor-*α* (PPAR-*α*) and hepatocyte nuclear factor-4*α* (HNF-4*α*), play important roles in regulation of hepatic lipid metabolism [73, 74]. However, excessive lipid accumulation in the liver is associated with the formation of ROS and release of proinflammatory cytokines [7, 75]. In such circumstance, hepatic accumulation of fat may impaire expression of zinc-finger proteins [75]. In addition, dysfunction of zinc-importing proteins by proinflammatory cytokines and oxidative stress reduces plasma zinc concentration, and zinc deficiency can thus exaggerate hepatic lipid accumulation and inflammatory response [73, 76]. Besides, dietary zinc deficiency has been shown to induce hepatic lipid peroxidation [77]. Also, reduced hepatic zinc levels have been correlated with the impaired liver function and regeneration in a great number of acute and chronic liver diseases [76], including NAFLD [74, 76]. Thus, as expected, zinc deficiency increased hepatic triglyceride, cholesterol, and levels of free fatty acids in association with downregulation of PPAR-*α* and lipid metabolism genes [73].

A growing body of evidence indicates that zinc offers a protection from acute and chronic liver injury, but its hepatoprotective properties have not been fully identified [73, 76, 78–80]. In current research zinc content in both rimonabant treated groups of mice was significantly increased compared to control values and mice fed on HFD (Table 2). This finding suggests that the pharmacological CB1 inactivation in hepatocytes in some manner may contribute to zinc protective effects, having in mind that this essential trace element plays a critical role in cellular integrity and biological functions [76].

Results in our study showed that copper and manganese content was significantly higher in HFD fed mice in comparison with control (Table 2). These findings may be related to published data that put into attention the development of NAFLD as a potential contributor to the disruption of homeostasis of copper, zinc, and selenium [74, 78]. Related to this, NAFLD in hepatitis C virus patients is associated with oxidative damage, likely due to imbalances in trace metals [74].

In our study significant increase in copper and manganese content was also observed in HFD fed mice after rimonabant treatment compared to mice fed on HFD, as well as in rimonabant treated mice in comparison with control group. On the other hand, in contrast to copper content, manganese content in rimonabant treated mice was decreased when compared to control values (Table 2). Judging from these findings, it seems that CB1 receptor blockade also may influence on hepatic copper and manganese status.

Rimonabant was the first drug developed to block CB1 receptors in the brain and in the periphery [81]. The effects of this drug on human body weight reduction and the metabolic profile were very encouraging [82] and so four related large clinical trials were launched to test the long-term effects of rimonabant, including its efficacy and safety [83–86]. However, rimonabant has subsequently been withdrawn due to serious side effects, such as nausea, arthralgia, diarrhea, dizziness, anxiety, depression, and suicidal ideation [87]. Although there is no future for rimonabant in human health, our study indicates that CB1 receptor antagonists may be useful in NAFLD treatment. It is still unclear which specific CB1-signaling pathways should be activated to produce the desired therapeutic effects versus which should be excluded to reduce the unwanted side effects [88].

In recent years, it becomes clear that G-protein-coupled receptor antagonists may have agonistic effects on other signaling pathways related to their receptors [88, 89]. Thus, this phenomenon, known as biased agonism, may be also responsible for beneficial effects of rimonabant in current research.

## 5. Conclusions

To date, no effective therapy has been proposed for patients with NAFLD. To our knowledge, this is the first study that investigates the effect of CB1 receptor blockade on oxidative/nitrosative liver injury in NAFLD. Based on our results, it can be noted the potential usefulness of CB1 blockade in the treatment of HFD-induced NAFLD, particularly due to attenuation of hepatic oxidative/nitrosative stress parameters and improvement of liver histology. However, further investigations regarding precise mechanisms by which HFD stimulate ECS production in the liver are required before moving toward clinical phase of investigation.

## Figures and Tables

**Table 1:** High saturated fat diet composition (MP Biochemical, CA, USA).

Nutrients	%
Casein purified	20
DL-methionine	0.30
Sucrose	30.58
Corn starch	20
Coconut oil	20
Alphacel, nonnutritive bulk	5
DL-*α*-tocopherol powder (250 IU/gr)	0.12
AIN-76 mineral mix	4

Total	100

**Table 2:** The effect of rimonabant on concentration of oligoelements in the liver.

Groups	Concentration of oligoelements (mg/kg)			
	Fe	Zn	Mg	Cu
C	82.90 ± 4.83	36.69 ± 5.02	1.27 ± 0.01	2.66 ± 0.15
R	88.09 ± 6.22	45.79 ± 6.16^∗∗^	0.06 ± 0.01^∗∗^	4.04 ± 0.48^∗∗^
HF	110.59 ± 10.07^∗∗^	29.10 ± 2.38^∗^	2.41 ± 0.23^∗∗^	4.33 ± 0.24^∗∗^
HFR	70.46 ± 10.07^##^	50.10 ± 8.65^##^	10.01 ± 1.99^##^	5.53 ± 1.38^##^

Fe—iron; Zn—zinc; Mg—manganese; Cu—copper.

C-control group fed with control chow diet for 20 weeks; R-group fed with standard chow diet and treated with rimonabant after 18 weeks; HF-group fed with high saturated fat diet for 20 weeks; HFR- group fed with high saturated fat diet and treated with rimonabant after 18 weeks.

Significance of the difference was estimated by using one-way analysis of variance (ANOVA) with Tukey's post hoc test (^∗^ *P* < 0.05, ^∗∗^ *P* < 0.01 vs. C, and ^##^ *P* < 0.01 vs. HF).
